# Overview on the Polyphenol Avenanthramide in Oats (*Avena sativa* Linn.) as Regulators of PI3K Signaling in the Management of Neurodegenerative Diseases

**DOI:** 10.3390/nu15173751

**Published:** 2023-08-27

**Authors:** Nitu L. Wankhede, Mayur B. Kale, Ashwini K. Bawankule, Manish M. Aglawe, Brijesh G. Taksande, Rashmi V. Trivedi, Milind J. Umekar, Ankush Jamadagni, Prathamesh Walse, Sushruta Koppula, Spandana Rajendra Kopalli

**Affiliations:** 1 Department of Pharmacology, Smt. Kishoritai Bhoyar College of Pharmacy, Nagpur 441002, Maharashtra, India; 2 Fortem Bioscience Private Limited, Bangalore 560064, Karnataka, India; 3 College of Biomedical and Health Sciences, Konkuk University, Chungju-si 27478, Republic of Korea; 4 Department of Bioscience and Biotechnology, Sejong University, Gwangjin-gu, Seoul 05006, Republic of Korea

**Keywords:** avenanthramide, AKT-protein kinase B, Alzheimer’s disease, Parkinson’s disease, oxidative stress, neuroprotection

## Abstract

Avenanthramides (Avns) and their derivatives, a group of polyphenolic compounds found abundantly in oats (*Avena sativa* Linn.), have emerged as promising candidates for neuroprotection due to their immense antioxidant, anti-inflammatory, and anti-apoptotic properties. Neurodegenerative diseases (NDDs), characterized by the progressive degeneration of neurons, present a significant global health burden with limited therapeutic options. The phosphoinositide 3-kinase (PI3K) signaling pathway plays a crucial role in cell survival, growth, and metabolism, making it an attractive target for therapeutic intervention. The dysregulation of PI3K signaling has been implicated in the pathogenesis of various NDDs including Alzheimer’s and Parkinson’s disease. Avns have been shown to modulate PI3K/AKT signaling, leading to increased neuronal survival, reduced oxidative stress, and improved cognitive function. This review explores the potential of Avn polyphenols as modulators of the PI3K signaling pathway, focusing on their beneficial effects against NDDs. Further, we outline the need for clinical exploration to elucidate the specific mechanisms of Avn action on the PI3K/AKT pathway and its potential interactions with other signaling cascades involved in neurodegeneration. Based on the available literature, using relevant keywords from Google Scholar, PubMed, Scopus, Science Direct, and Web of Science, our review emphasizes the potential of using Avns as a therapeutic strategy for NDDs and warrants further investigation and clinical exploration.

## 1. Introduction

Since ancient times, cultivated oats have been an important cereal crop for human consumption worldwide. Recently, interest in oats has been increased due to their nutritional properties and associated health benefits [1]. In the world grain ranking, oats were placed sixth for their nutritional values and regarded as most suitable for cultivation under variable climatic and soil conditions [2,3]. Cereal oats contain numerous bioactive compounds with high nutritive values including cellulose, arabinoxylan, β-glucans, proteins, unsaturated fatty acids, vitamins, minerals, phenols, and dietary fiber [4,5]. Studies suggest that the regular consumption of oat grains is associated with a lower risk of various chronic diseases [6,7]. Meta-analysis studies also revealed that regular oat intake lowers blood cholesterol and improves insulin sensitivity and post-prandial glycemic control [6,7]. The water-soluble β-glucan contained in oat seeds mainly lowers cholesterol levels, leading to a reduced risk of heart disease in humans [8,9]. Further, pharmacological studies indicate that the polyphenolic active constituents in oats exhibit strong anti-inflammatory, anti-bacterial, anti-carcinogenic, cytotoxic, and anti-proliferative properties [4,10]. For this reason, the human demand for oat consumption has been drastically increased.

In the central nervous system (CNS), different signaling pathways regulate various cellular activities and physiological functions. In particular, the protective roles of the phosphoinositide 3-kinase (PI3K) signaling pathway and protein kinase B (AKT) have been widely reported in neurodegenerative conditions. The PI3K/AKT signaling pathway is functional in many CNS processes such as synaptic plasticity, neurogenesis, proliferation and differentiation, aging, and neuronal autophagy [11]. In addition, PI3K also plays a predominant role in molecular trafficking [12]. The PI3K/AKT pathway constitutes a major signaling cascade, which consists of PI3K, a multifaceted protein, and its downstream molecules including glycogen synthase kinase-3 beta (GSK-3β), the mammalian target of rapamycin (mTOR), and the nuclear factor erythroid 2–related factor (Nrf) [13].

In a growing number of studies, neuroprotective agents from natural products are involved in targeting PI3K/AKT signaling, thus contributing to the prevention and treatment of neurodegenerative diseases (NDDs) including Alzheimer’s disease (AD) and Parkinson’s disease (PD) [14,15]. Although great efforts have been made to understand the pathogenesis of NDDs and the design of an effective treatment to delay progression, there is still no potential therapy available. Thus, the interest in derived natural compounds with potential benefits in CNS disorders has been substantially growing. The mechanisms of action of natural compounds are variable, suggesting that they could be highly effective and specific in improving neuroprotective capacity.

**Figure 1:**
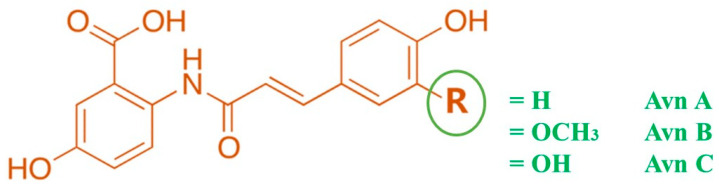
Structure of avenanthramides.

Avenanthramides (Avns, Figure 1) are the major water-soluble phenolamides obtained exclusively from oats. Several chronic conditions, including cardiovascular disease, cancer, diabetes, and neurological and skin disorders, are among those for which the therapeutic potential of Avns has been widely reported [1,7]. Recently, Avns and their derivatives were reported to possess neuroprotective effects in experimental models and also improve AD and PD pathologies including memory and behavioral impairments [16,17,18,19].

Among the 25 polyphenolic Avns identified, avenanthramide c (Avn-c), an amide conjugate of anthranilic acid and hydroxycinnamic acid, was found to be protective in experimental models of cerebral ischemia [10,20,21]. However, the roles of Avns and their derivatives against NDDs and their mechanistic basis have not been fully understood. In the present review, we highlight the potential of Avns as novel therapeutics for the treatment of NDDs and the influence of Avns on the regulation of the PI3K signaling pathway in the pathophysiology of NDDs including AD and PD. The relevant literature, using the keywords “avenanthramide”, “PI3K/AKT”, “neurodegenerative diseases”, “AD”, and “PD” from Google Scholar, PubMed, Scopus, Science Direct, and Web of Science were selected. The most appropriate and recent articles were prioritized based on the purpose of this review.

## 2. Modulation of PI3K Signaling Pathway in NDDs

It is well documented that the dysregulation of PI3K signal transduction significantly contributes in the pathogenesis of various NDDs including AD, PD, and Huntington’s disease [22]. Further, signal transduction involving AKT along with PI3K is known to mediate neuronal survival. Therefore, understanding the role and regulatory aspects of the PI3K and AKT pathways might help in the development of suitable therapeutic agents against NDDs targeting PI3K/AKT signaling. Although the roles of PI3K/Akt and the mechanisms involved in NDDs were thoroughly reviewed (Rai et al. 2019) [23], in the following sections, the key aspects of PI3K/AKT activation and other related signaling molecular pathways in relation to NDDs are discussed.

### 2.1. PI3K

According to the structure, regulation, and substrate specificity, PI3Ks have been divided into three subcategories: classes I, II, and III [24]. Among these, the class I isoform has been studied extensively, which is activated by surface receptors composed of regulatory and catalytic subunits. These are further subcategorized into class IA and class IB based on the mode of regulation. Class II PI3K enzymes require an additional signal for activation, whereas class III PI3K enzymes are vacuolar protein sorting 34 (Vps34) required mainly for membrane trafficking [25].

### 2.2. AKT-Protein Kinase B (PKB)

AKT has been considered as a core effector of the PI3K downstream signaling pathway. AKT is a serine/threonine kinase, categorized into three homologous isoforms, AKT1/PKBα, AKT2/PKBβ, and AKT3 [14,23]. It consists of three different domains, including the pleckstrin homology (PH) domain at the N-terminal, which is responsible for membrane translocation after activation by PI3K; the central fragment; and the regulatory domain at C-terminal, which contains the phosphorylation site required for the activation of AKT. Among the isoforms, AKT3/PKBγ is crucial for the development of the brain and microglial survival.

### 2.3. Activation of PI3K/AKT Pathway

The signaling pathway begins with the interaction between the ligand and transmembrane receptor, mainly tyrosine kinase (RTK), which results in receptor dimerization and the autophosphorylation of the intracellular tyrosine domain, leading to the recruitment of PI3. The P85, a regulatory domain in class I PI3K, binds to the phosphorylated tyrosine residue following the recruitment of p110, a catalytic domain on PI3K that is responsible for the complete activation of the PI3K enzyme. The p110 also recruits the inactive AKT and phosphatidylinositol-dependent protein kinase 1 (PDK1) from the cytoplasm on the cell membrane that brings conformational changes in the AKT, exposing the phosphorylation site on serine (473) and threonine (308). The activated PI3K also phosphorylates phosphatidylinositol 4,5 biphosphate (PtdIns,4,5, P2) to phosphatidylinositol 3,4,5 triphosphate (PtdIns,3,4,5, P3), which becomes dephosphorylated with phosphatase and tensin homologue on chromosome 10 (PTEN), which regulates the inactivation of AKT [26].

The phosphorylated PI3K/AKT-Ser473 forms are complex with a wide range of downstream signaling molecules including GSK-3β, mTOR, ERK, NF-κB, Hsp, etc., to execute diverse cellular activities [14,27]. The downstream target mTOR regulates the metabolism. The activation of PI3K/AKT also inhibits the process of apoptosis by interacting with different signaling molecules including the Bcl2 antagonist of cell death (Bad), the bcl-2-like protein 11 (BIM), and caspase-9. NF-κB, one of the downstream modulators of PI3K/AKT, regulates the inflammatory response by modulating the expression of inflammatory markers IL-1β, IL-6, TNF-α, iNOS, and COX-2. At the resting state, the activity of NF-κB is repressed by inhibitory kappa B (IκB). The PI3K/AKT interacts with nuclear factor erythrocyte two related factors (Nrf2) to regulate oxidative stress [14]. In NDDs including AD and PD, the PI3K/AKT signaling is altered, resulting in the disruption of cellular function including autophagy and synaptic plasticity, thus indicating that targeting the PI3K/AKT pathway or its downstream regulator could be a novel strategy to treat NDDs.

## 3. PI3K/AKT Pathway in Neurodegeneration

NDDs are the category of conditions with the selective and progressive loss of structure or function of neurons. These disorders are characterized by the gradual degeneration of neurons, resulting in behavioral, learning, emotional, and cognitive abnormalities [28]. The prevalence report suggests a steady increase in the number of people suffering from neurodegenerative conditions. The treatment is targeted to alleviate symptoms and to prevent progression to improve the quality of life. Many studies have been carried out to understand the pathophysiological cause of the progressive neurodegeneration associated with AD and PD, which highlighted several possible targets for the development of neuroprotective strategies for effective treatment [29]. In this section, we discuss the evidence for the involvement of PI3K/AKT downstream targets in AD and PD. In addition, we highlight the potential targets of the PI3K/AKT pathway for the treatment of NDDs.

Reactive oxygen species (ROS) are produced in normal physiological settings as a bioproduct of cellular metabolism. However, ROS levels are firmly regulated via redox homeostasis. The production of ROS is increased in certain pathophysiological conditions due to several risk factors, including environmental stress, mutation, or genetic factors. Failing to regulate the excessive production of ROS causes structural and functional problems and the loss of cellular function. Neurons are particularly vulnerable to oxidative stress conditions due to a high consumption of oxygen, low antioxidant levels, high polyunsaturated membrane fatty acids, as well as a post-mitotic high accumulation of oxidized molecules [30]. In NDDs such as AD and PD in particular, oxidative stress has been considered to be a crucial risk factor. Thus, increased oxidative stress markers as well as deficient antioxidant defense systems are considered to be the common hallmarks in these conditions [30,31,32,33].

One of the main neuroprotective factors that regulate antioxidant and anti-inflammatory genes is Nrf2. Nrf2 modulates oxidative stress by regulating the synthesis of antioxidant enzymes through binding with the antioxidant response element (ARE). Under a physiological setting, the activity of Nrf2 is repressed by a protein, Kelch-like ECH-associated protein 1 (Keap1), which is an adaptor for the E3 ligase in the ubiquitin-proteasome pathway [34,35]. However, under stressful conditions, Keap1 is inactivated due to post-translational modification, leading to migration and the accumulation of active Nrf2 inside the nucleus, leading to heterodimerization with small protein designated as sMaf, which stimulates the expression of antioxidants through binding at ARE on the target gene [33,36]. Besides Keap1, the activation of the PI3K/AKT pathway can also indirectly regulate Nrf2-ARE signaling through a serine/threonine protein kinase designated as glycogen synthase kinase 3β (GSK-3β) via phosphorylating Nrf2 [37,38]. Mounting evidence suggests that the negative regulation of GSK-3β via PI3K/AKT signaling can regulate the activity of Nrf2 through stabilizing and regulating the gene expression of Nrf2 [39,40,41]. Under physiological conditions, the activation of PI3K/AKT inhibits GSK-3β through phosphorylation at Ser21-GSK-3α or Ser9-GSK-3β [42].

Several studies investigated that the PI3K/AKT/Nrf2/GSK-3β pathway has been impaired in AD patients and pre-clinical mouse models [43,44,45,46]. Oxidative stress in AD causes the downregulation of phosphorylated PI3K, which results in the inactivation of the PI3K/AKT pathway, leading to GSK-3β activation, which translocates Nrf2 from the nucleus into cytosol, resulting in low levels of antioxidant enzymes [47,48,49,50]. In addition, amyloid-β oligomers were found to activate GSK-3β, blocking PI3K/AKT/mTOR signaling, which increases the phosphorylation of Tau, inducing neurofibrillary tangle formation, which is a pathophysiological hallmark in AD. These findings suggest the involvement of different downstream molecules of PI3K/AKT in the pathophysiology of AD.

Oxidative stress-induced neuronal death also contributes to the pathology of PD. PI3K/AKT signaling influences oxidative stress by modulating downstream molecules such as GSK-3β and mTOR. The abnormal expression of GSK-3β has been reported in PD. Several findings indicate that Nrf2 signaling is compromised with age; thus, reduced Nrf2 expression in different brain regions has been associated with increased age [51,52], though the expression of Nrf2 has been suggested to be restricted in astrocytes in substantia nigra (SN), which is still sufficient to protect neurons from 1-Methyl-4-phenyl1,2,3,6-tetrahydropyridine (MPTP)-induced neurotoxicity [53]. In addition, data from post-mortem brains indicated Nrf2 dysfunction in PD [52,54]. Also, the levels of downstream targets in PI3K/AKT/mTOR signaling were found to be significantly reduced in PD patients. In PD, the imbalance of oxidative stress has been reported due to disrupted FoxO3a, a downstream target of mTOR [55,56]. Thus, the molecules that activate the PI3K/AKT pathway through activation of p-GSK-3β (Ser9) or mTOR can prevent oxidative-stress-induced neuronal death, which can be beneficial in preserving neuronal structure and function.

## 4. Neuroprotective Role of Avns

Avns derived from oats have been shown to exert neuroprotective effects by modulating signaling pathways involved in cell survival, neuronal growth, synaptic plasticity, apoptosis, and neuroinflammation. These mechanisms can enhance neuronal resilience and support the maintenance of cognitive function. Oat polyphenols have been well reported for their pharmacological benefits including antioxidant activities. The polyphenolic combination with different groups such as phenolamide tends to potentiate its action [57,58,59]. Avns have been found to exhibit antioxidant and anti-inflammatory effects in the CNS, which can be beneficial for reducing stress and neuroinflammation associated with various neurological conditions.

In a preclinical study, Avns was found to reduce the area of tissue damage and improved the neurological outcomes following stroke in the middle cerebral artery occlusion (MCAO) in mice models [17]. These effects were attributed to the ability of Avns to enhance blood flow, inhibit inflammatory responses, and protect against oxidative damage. Furthermore, Avns have been found to promote neurogenesis and enhance synaptic plasticity, which are crucial for brain repair and functional recovery after stroke [16]. While the research on Avns in neurological conditions is still emerging, the available evidence suggests their potential as neuroprotective agents. In the following sections, we discuss the beneficial roles of Avns against NDDs including AD and PD pathology.

### 4.1. Alzheimer’s Disease

Globally, most geriatric people are suffering from this common condition, which is associated with dementia, memory loss, and cognition impairment [60]. According to the annual report of the Alzheimer’s Association, about 6 million Americans belonging to the age group of 65–75 years most commonly have AD, and the incidence will increase day by day. Nerve cell destruction, extracellular amyloid plaques, and neurofibrillary tangles (NFTs) within the cell seem to be the markers of this complex NDD. These plaques are composed of amyloid beta (Aβ), a cleavage by-product of the amyloid precursor protein (APP) [61]. Moreover, the gradual formation of oligomers, fibrils, and insoluble amyloid plaques from Aβ monomers results in a reduction in the plasticity of neurons in the synaptic domain [62]. In addition, tau protein has been hyperphosphorylated, which forms NFTs. In healthy conditions, tau encourages microtubule stabilization. But as paired helical filaments connect, hyperphosphorylated tau builds up and eventually produces NFTs. The accumulation of Aβ leads to the dysregulation of synaptic and neuronal activities, which further generates intracellular conditions for NFT production and ultimately results in neuronal death and the disruption of neurotransmitter functions [63].

The antioxidant potential of Avns has been confirmed through a variety of in vivo and in vitro studies. The hydroxy and amide group in its structure are mainly responsible for scavenging free radical species formed during a variety of physiological as well as pathological conditions. This phenolamide is also able to induce the synthesis of a variety of cytoprotective enzymes. In addition, Avn contains an unsaturated amide group, which mainly interacts with the cysteine residue in Keap1, thus effectively preventing the phosphorylation and accumulation of Nrf2 in the cytosol and promoting the upregulation of the transcriptive action of Nrf2 [64,65].

Synaptic dysfunction has a major contribution to memory deficits in AD pathophysiology. Thus, molecules that strengthen the synapse could be beneficial for the treatment of AD. Alteration in synaptic plasticity due to the accumulation of amyloid-β, leading to memory loss and dementia, is a key feature of AD. The methanolic extract of Avns was found to restore and alter long-term potentiation in CA3 to the CA1 region of the hippocampus in the Tg2576 AD mouse brain [19]. In AD, amyloid-β aggregates were found to act through activating GSK3β of the PI3K/AKT pathway [66,67]. The study revealed that Avn-C, in particular, modulates the S9-GSKβ, leading to an improvement in brain function that was altered due to amyloid-β accumulation. The inhibition of S9-GSKβ effectively upregulates Nrf2-ARE activity, which may be responsible for the neuroprotective action of Avn-C in Tg2576 mice [19].

**Figure 2:**
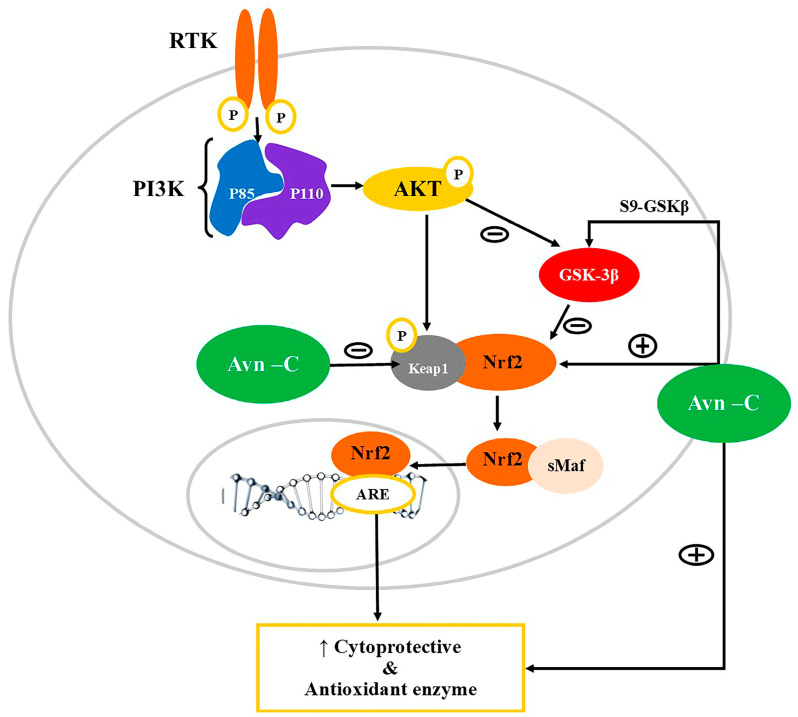
Modulation of PI3K/AKT/Nrf2/GSK-3β signaling pathways by Avn. PI3K: Phosphatidylinositol-3-kinases (PI3Ks); AKT: Ak strain transforming-Phosphokinase B; GSK3β: Glycogen synthase kinase 3β; Nrf2: Nuclear factor erythroid 2-related factor 2; ARE: Antioxidant response element; Keap1: Kelch-like ECH-associated protein 1; RTK: Receptor tyrosine kinase. Oxidative stress limits the action of PI3K/AKT, and thereby activates GSK3β, thus inhibiting Nrf2 translocation into the nucleus, leading to downregulation of expression of cytoprotective enzymes. Avn-C acts as an electrophile that interacts with Keap, resulting in loss of inhibitory control and translocation of Nrf2 into the nucleus and activating ARE for transcription of the antioxidant enzyme. Avn-C can also activate Nrf2 indirectly by inhibiting GSK3β.

In addition, it was also reported that Avn-C acts as an amyloid inhibitor, thereby preventing Aβ protein aggregation [68]. The study showed that Avn-C improved the memory deficits associated with the Aβ and was reported to strengthen the synapse indicated via an improvement in LTP. The study also revealed that Avn-C effectively reduced the caspase-3 concentration and thereby inhibited the activation of pro-inflammatory markers, and in contrast, increased the levels of anti-inflammatory markers. Further, Avn-C activated the S9GSK3β and improved the antioxidant defense through the activation of Nrf2 in a different model of AD [69]. A possible role of Avn in the modulation of the PI3K/AKT/Nrf2/GSK-3β signaling pathways is shown in Figure 2.

### 4.2. Parkinson’s Disease

PD is the second most common neurodegenerative condition, affecting 1% of the old age population. Clinical presentation in PD is associated with motor disturbances including bradykinesia, rigidity, and tremors [70]. Majorly, PD is characterized by a progressive loss of dopaminergic neurons in SN, but the neuropathology indicates a widespread association between different regions of the brain, where the involvement of SN occurs in the middle stage of the disease. The pathological hallmark of the disease involves the deposition of Lewy bodies, which are round eosinophilic protein aggregates composed of α-synuclein (α-syn) and synphililin-1 [71,72]. A variety of factors are responsible for the alteration in transcription in the sporadic form of PD such as environmental factors, oxidative stress, exposures, and aging [73,74], whereas the familial type of PD is associated with a mutation in genes encoding α-syn [73,74,75,76,77]. Studies have suggested that among the list of factors, oxidative stress and ROS are mainly responsible for neuronal loss in the SN region in the PD pathophysiology [78,79]. Further, previous reports also indicated that several flavonoids and polyphenolic compounds that form natural products exhibited neuroprotective effects by suppressing neuroinflammation, oxidative stress, and improved cognition by regulating the PI3K/AKT signaling pathways in PD models [80,81,82].

In a recent study, Bisavenanthramide-B (Bis-B), a synthetic analog of Avn-C, appears to protect from oxidative stress [83]. The molecule was discovered as a product formed during the reaction of Avn with ROS. Bis-B contains an electrophilic group designated as a Michael acceptor, which is capable of covalently conjugating with cysteine residues of Keap1, thereby inducing conformational changes and leading to the translocation of Nrf2 in the nucleus, which results in cytoprotective gene expression through Nrf2-ARE interaction [83].

The oxidative stress induced via dysfunctional mitochondria has been a well-known pathology in NDDs. These synthetic Avns have also shown neuroprotective effects against rotenone/oligomycin-induced oxidative damage, which was considerably similar to the PD pathology. Thus, Bis-Avns could be the effective therapeutic approach in preventing selective SN dopaminergic degeneration in PD. Further, the same study revealed that Avn has excellent neuroprotective action through directly and indirectly scavenging ROS via Nrf2 stimulation against okadaic-acid-induced Tau hyperphosphorylation and oxidative stress in SH-SY5Y cells, which has been linked critically with the neurodegenerative tauopathies in AD and PD [84,85]. In addition, Avn-C can directly protect from oxidative stress through the activation of Nrf2-ARE [18]. The Avn-2c isoform efficiently translocates Nrf2 into the nucleus in PC12 cells and thereby stimulates the expression of cytoprotective enzymes, and thus could be beneficial in the treatment of NDDs including PD. The available literature on the therapeutic potential of Avn and its derivatives are shown in Table 1.

## 5. Modulation of Other Targets by Avns

Phytochemicals are mainly associated with neuroprotective benefits against numerous risk factors including chemical toxicity and oxidative stress in neurodegenerative conditions. Oat extract enriched with Avns possesses a neuroprotective role against toxicity and oxidative stress induced by titanium dioxide nanoparticles (Tio2NPs) in rats [86]. Biochemical and histopathological studies revealed that the combination of Avns with thymoquinone exerts antioxidant and anti-inflammatory action, thus preventing neuronal degeneration induced with Tio2NPs [86]. The Avns improved the deleterious effect by altering the oxidative stress and improving antioxidant concentration.

Numerous studies suggested that the levels of pro-inflammatory markers including TNF-α, IL-1β, and IL-6 were significantly increased in the cerebrospinal fluid of AD patients and were correlated with impairment in LTP in animal models of AD [87,88,89]. Thus, the inhibition of the inflammatory pathway would be an effective approach to improve memory impairment. Ramasamy et al. reported that treatment with Avn-C significantly improved memory impairment in Tg2576 and 5XFAD mice brains [16]. In addition, the study also reported that Avn-C reduced the level of inflammatory markers, which could be a potential mechanism of action. Several studies also suggested that the levels of TNF-α were significantly reduced with Avn-c treatment [90,91,92].

**Figure 3:**
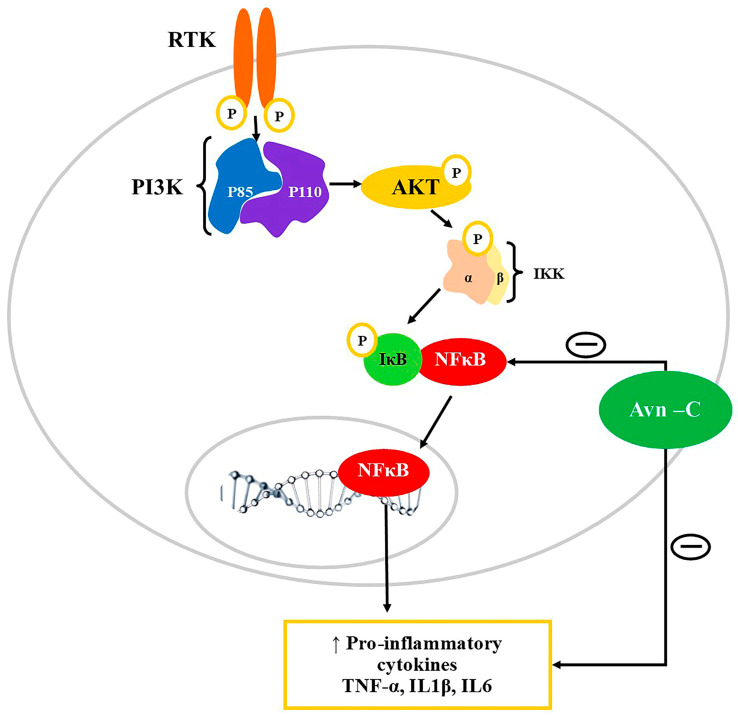
Modulation of PI3K/AKT/NF-κB signaling pathway by Avn-c. PI3K: Phosphatidylinositol-3-kinases (PI3Ks); AKT: Ak strain transforming-Phosphokinase B; RTK: Receptor tyrosine kinase; NF-κB: Nuclear factor kappa B; IKK: Inhibitor of NF-κB kinase; IκB: Inhibitor of nuclear factor-κB; TNFα: Tumor necrosis factor; IL-1β: Interleukin 1β; IL6: Interleukin 6. Avn-C downregulates the expression of inflammatory cytokines by inhibiting NF-κB and prevents its translocation into the nucleus thereby. Avn-C can directly inhibit the action of inflammatory mediators including TNFα, IL6, and IL1β.

In addition, Avns were found to reduce inflammation via altering the TNF-α concentration. The Avns were found to limit the TNF-α-induced neurogenic inflammation via modulating the NF-κB signaling and IL-8 levels [90,93]. Thus, the anti-inflammatory mechanism of Avn-c could be beneficial in the treatment of neurodegenerative conditions (Figure 3). The levels of the neurotransmitter acetylcholine are reduced due to hydrolysis due to the overstimulation of acetylcholinesterase in AD patients. Thus, targeting acetylcholinesterase could be beneficial in AD treatment [94]. Avn was also found to inhibit acetylcholinesterase and can thus improve memory in neurodegenerative conditions like AD [83,95].

## 6. Future Perspectives

Oats are a useful grain with distinct constituents and a unique source of Avns possessing abundant nutritional benefits. Recently, focus has been given to the biological activities of naturally produced Avns as well as their derivatives in the treatment of NDDs. Avenanthramides’ anti-inflammatory effects make them attractive candidates for mitigating neuroinflammation and its detrimental impact on the progression of these diseases. Future studies could focus on investigating their specific anti-inflammatory mechanisms and assessing their effectiveness in reducing neuroinflammation. Further, the antioxidant properties of Avns make them potential candidates for managing oxidative stress and preventing neuronal damage. Research could explore their potential in slowing or halting the progression of NDDs including AD and PD.

While Avns show promise, it is important to note that further research is necessary to fully understand their mechanisms of action, optimal dosages, and long-term effects in the context of NDDs. Further, the beneficial effects of Avns using synthetic Avn analogs and Avns obtained via recombinant techniques should also be explored in the treatments of NDDs. For this purpose, collaboration between researchers and clinicians will be crucial in translating the potential of Avns into effective therapeutic interventions for NDDs.

## 7. Conclusions

The PI3K/AKT signaling pathway in NDDs has been proven to be a successful hypothesis for the development of an effective treatment approach and more detailed research is being conducted to understand the involvement of downstream targets for the treatment of NDDs. Avn holds potential as a treatment for NDDs caused by oxidative stress. The protective effects of Avns are mediated mainly through the activation of the Nrf2, a downstream target of PI3K/AKT, which upregulates transcription genes encoding antioxidant enzymes. Avn-C and its synthetic analog were also found to modulate other targets PI3K/AKT including GSKβ and NF-κB in CNS. The molecule also possesses anti-inflammatory and anti-apoptotic action in wide pathological states including cancer and skin inflammation, indicating that its mechanism could be beneficial for the treatment of NDDs. In conclusion, the need for in depth pre-clinical studies in various models of neurodegeneration and clinical exploration to elucidate the specific mechanisms of Avn action and its potential interactions with other signaling cascades involved in neurodegeneration is quite essential.

## Figures and Tables

**Table 1:** Therapeutic potential of avenanthramides in neurodegenerative disease.

No.	Study Design	Mechanism	Methodology	Reference
1	Electrophysiological study on hippocampal LTP in Tg2576 male mice	Alteration in p-GSK-3β-S9 levels and reduction in Caspase 3	Avn-C extracted from germinated oats using column chromatography	[19]
2	Evaluation of neuropathologies and behavioral impairments associated with AD	Modulates p-GSK-3β-S9 Reduced caspase 3 and neuroinflammation Binds to α1A adrenergic receptors to stimulate phospho-AMPK levels	For electrophysiological studies on the hippocampal slices of WT, Tg2576 and 5XFAD mice were treated with Aβ42 oligomers in the presence of Avn-C (10, 25, and 50 μM)	[16]
			Tg2576 and 5XFAD mice were administered with Avn-C (2, 4, and 6 mg/kg. p.o.) for evaluation of long-term potentiation	
3	Effect on protein aggregation using spectroscopy techniques	Inhibition of BSA oligomerization showing anti-amyloid effect	Protein aggregation in bovine serum albumin was initiated by incubating the protein monomer at an elevated temperature and the aggregation kinetics was monitored by incubating with Avn-C (100, 250, and 500 μM) and was analyzed using ThT-fluorescence assay	[68]
4	Evaluation of bisavenanthramide analogues of Nrf2 inductors and neuroprotectors in in vitro models	Nrf2-ARE-dependent protein expression	The antioxidant activity was measured using DPPH scavenging assay and FRAP method, and AChE inhibition assay was performed. Neuroprotection potential against tau hyperphosphorylation was evaluated using SH-SY5Y cell line	[83]
5	Evaluation of cytoprotective activity against oxidative-stress-induced PC12 cell injuries	Activating Nrf2-ARE pathway	Rat PC12 were used to study antioxidant effect of Avns. The antioxidant and cytoprotective activities of Avn-2c, Avn-2f, and Avn-2p were measured in vitro using the ABTS•+ and DPPH scavenging assay, MTT, and LDH release assay	[18]
6	Cognitive dysfunction induced by repeated propofol anesthesia in aging rats	Activating Nrf2/ARE pathway	Aging rat model was established by continuous 200 mg/kg propofol anesthesia	[69]
7	Protective effect on titanium dioxide nanoparticles induced neurotoxicity in SD rats	Decreases oxidative stress and TNF-α Increases the total antioxidant and GSH levels	TiO2 NPs (150 mg/kg b.w.) was administered orally for six weeks and Avn was administered daily at a dose of 20 mg/kg via gastric tube	[86]

Abbreviations: Avns, Avenanthramides; LTP, Long-term potentiation; AMPK, AMP-activated protein kinase; GSK-3β, Glycogen synthase kinase 3 beta; TNF-α, Tumor necrosis factor alpha; WT, Wild type; SD, Sprague Dawley; GSH, Glutathione; Nrf2/ARE, NF-E2-related factor 2/antioxidant response element; TiO2 NPs, Titanium dioxide nanoparticles; BSA, Bovine serum albumin; PC12, Pheochromocytoma cells; DPPH, 2,2-diphenylpicrylhydrazyl; MTT, 3-(4,5-Dimethylthiazol-2-yl)-2,5-Diphenyltetrazolium Bromide; LDH, Lactate dehydrogenase; SH-SY5Y, Neuroblastoma cell line; ABTS, 2,2′-azino-bis (3-ethylbenzothiazoline-6-sulfonic acid; AChE, Acetylcholinesterase; FRAP, Ferric reducing antioxidant power; ThT, Thioflavin T fluorescence.

## Data Availability

Not applicable.
